# Assessment of Local and Metastatic Recurrence Following Robot-Assisted Radical Prostatectomy by Margin Status Using PSMA PET/CT Scan

**DOI:** 10.3390/cancers18010043

**Published:** 2025-12-23

**Authors:** Thomas Edward Ahlering, Yeagyeong Hwang, Michael Matthew Lee, Joshua Tran, Anders David Jens Carlson, Levon Kazarian, Karren Liang, Whitney Zhang

**Affiliations:** Department of Urology, University of California, Irvine Medical Center, Orange, CA 92868, USA; yeagyeoh@hs.uci.edu (Y.H.); michaml4@uci.edu (M.M.L.); andersdc@uci.edu (A.D.J.C.); lkazaria@uci.edu (L.K.);

**Keywords:** metastatic prostate cancer, radical prostatectomy, PSMA PET/CT, local neoplasm recurrence, surgical resection

## Abstract

**Background**: Local recurrences following radical prostatectomy (RP) are typically attributed to incomplete surgical resection or positive surgical margins (PSMs). Yet approximately 70% of men with PSMs never experience BCR. Prostate-specific membrane antigen PET scans (PSMA PET) are useful in detecting the incidence and location of recurrence sites. This study explores the relationship between margin status and local and metastatic recurrences using PSMA PET scans. **Methods**: A retrospective study was conducted with prospectively collected data following RARP with BCR in 159 men undergoing PSMA PET (2017–2023). The primary outcome compared risk and location of recurrences between NSM vs. PSM. A total of 13 cases (8%) had “equivocal” PET scan findings which were assessed first as all positive and then all negative. **Results**: Of 159 men with BCR undergoing PSMA PET scans, 101 (63.5%) had NSMs and 58 (36.5%) had PSMs. Assuming all 13 “equivocal” scans were positive, the risk of a positive PSMA PET is NSMs vs. PSMs (73% vs. 69% *p* = 0.56). Local recurrence rates did not differ significantly (NSMs 39.2% vs. PSMs 45% *p* = 0.55), nor did lymph nodes (NSMs 61% vs. PSMs 58% *p* = 0.73) or bone lesions (NSMs 16.2% vs. PSMs 22.5% *p* = 0.41). Multivariate regression analysis showed that margin status was not a predictor of local recurrence (OR 1.40; 95% CI [0.65, 1.54]; *p* = 0.382). **Conclusions**: Local recurrence occurs at about the same rate independent of margin positivity status, suggesting that local recurrences appear to be more closely related to metastatic dissemination, not incomplete resection. These findings question the oncologic rationale for wider resections at the expense of functional outcomes.

## Simple Summary

After radical prostatectomy (RP), many clinicians assume that having “negative surgical margins,” (meaning all cancer was fully removed) lowers the chance of cancer returning locally. However, our study of 159 men who developed a rise in PSA after RP challenges this long-held belief. Using PSMA PET/CT scans, which is one of the most sensitive imaging tools for detecting recurrent prostate cancer, we found that men with positive and negative surgical margins had nearly identical rates of local recurrence, as well as similar rates of lymph node and bone metastases. Our results illustrate that pursuing wider resections to achieve negative margins, often at the cost of urinary continence and erectile function, may not improve outcomes. Earlier detection and timely evaluation with PSMA PET may be more important for long-term cancer control than margin status alone.

## 1. Introduction

Prostate cancer (PC) is the most common non-cutaneous cancer in the United States and is the second leading cause for cancer-related mortality in men. According to the American Cancer Society, nearly one in eight men will be diagnosed with PC during their lifetime, and one in forty-one will die from the disease [1]. Following the 2012 U.S. Preventive Services Task Force recommendation against prostate-specific antigen (PSA) screening, the incidence of PC initially declined significantly [2]. However, due to the long natural history of PC, the downstream effects of reduced screening, shown as distance disease progression and prostate cancer-specific mortality (PCSM), are becoming more obvious and problematic [3,4,5,6,7]. Unlike most other malignancies, PC is vastly different in that it progresses slowly yet persistently, both before and after treatment intervention. It is well known that most men with recurrent disease will die with PC rather than of it, even in the metastatic setting [8]. This is in contrast to most other cancers, where recurrence following surgical resection typically results in rapid mortality if not cured with secondary intervention. For PC, metastatic recurrences following radical prostatectomy (RP) are rarely curable. However, systemic therapies remain remarkably effective at controlling but not eradicating disease. Even among men with high-risk PC, mortality is only approximately 50% at 29 years following RP [9].

Another significant factor in PC prognosis is positive surgical margins (PSMs). In most cancers, including those in the breast, bladder, and colon, recurrence is an adverse outcome leading to worse survival, high rates of metastasis, and cancer-specific mortality [10,11,12], and it is most notably predicted by the presence of a positive surgical margin [13,14]. In PC, however, the prognostic value of surgical margin status remains a subject of debate. Though some report higher rates of biochemical recurrence (BCR) predicted by PSM, the independent association between PSM and PCSM has not been reliably established. In fact, Pellegrino et al. [15] recently noted in 2023 that a solitary PSM, regardless of size, did not confer increased risk of PCSM as compared to negative surgical margins (NSMs). Subsequently, it was shown that the BCR rate for solitary PSMs at 10 years after RP was only 30%, raising the question of why 70% of men with “incomplete resection” had no recurrence. The logical explanation for the lack of PSA progression in the face of a PSM would suggest that PC cells at the pathological margin must simply senesce and die 70% of the time [16,17]. Prostate-specific membrane antigen PET/CT scanning has revolutionized PC management. With high sensitivity and specificity for detecting and localizing local and distant metastases even at low PSA levels, PSMA PET/CT offers an unprecedented opportunity to evaluate the patterns of disease recurrence [18,19]. This study aims to further examine the PSMA PET scan results of our first 159 men with BCR to compare primarily PSMs versus NSMs.

## 2. Materials and Methods

This was a single-center retrospective analysis of 159 patients with BCR following RARP by a single surgeon (TA) who had a PSMA PET/CT scan between 2017 and 2023. Pelvic lymph node dissection (PLND) was not routinely performed at the time of RARP in this cohort. When indicated, it was performed in the salvage setting based on PSMA PET/CT-identified nodal recurrence. PSMA PET/CT was performed in response to rising PSA levels (approximately 0.7–1.0 ng/mL) and/or in selected patients with higher preoperative risk, as part of routine clinical care rather than at a fixed postoperative interval. A total of 90% of the PSMA PET scans were performed and interpreted at the University of California Los Angeles Medical Center using ^68^Gallium-labeled PSMA radiotracer (University of California, Los Angeles, Los Angeles, CA, USA) [20]. BCR was defined as two PSA levels > 0.2 ng/mL post-RARP. Since 13 (8%) of the 159 scans had “equivocal” PSMA-PET/CT scan findings, we performed two analyses: one assuming all equivocal findings were positive and, in a secondary supplemental analysis, one assuming all equivocal findings were negative. PSMA PET/CT findings were classified based on finalized pathology-issued imaging reports. Equivocal findings were interpreted according to radiologic descriptors provided in the report, with lesions demonstrating focal uptakes above background activity at anatomically plausible sites classified as positive. Equivocal lesions were defined as sites of low-level focal uptake that did not definitely exceed physiological background activity and/or lacked a clear anatomic correlate on CT. All equivocal cases were reviewed in consensus by board-certified pathologists with expertise in molecular imaging, minimizing inter-reader variability. The study was conducted under an approved institutional review board protocol at the University of California, Irvine (HS#1998-84).

Demographic and clinical variables, including age, PSA levels at time of PSMA-PET/CT scan, pathological stage, Gleason grade group, and surgical margin status, were collected from the patients’ medical records. Patients were stratified based on margin status (positive vs. negative) and scan result (positive vs. negative findings). To assess the oncological value of complete local resection, the primary outcome was to compare the rate and location of recurrences between men with NSMs and PSMs. Local recurrence was defined as PSMA PET/CT-avid uptake localized to the prostate bed and/or seminal vesicle (SV) remnants. Uptake was required to be focal and greater than surrounding background activity. No fixed SUV threshold was applied, consistent with clinical practice. Benign postoperative changes were carefully considered, particularly in the prostate bed. Uptake patterns suggestive of postoperative inflammation were not classified as positive. Lymph node metastasis and bone metastasis were defined as one or more lymph nodes or bones with PSMA PET/CT uptake, respectively.

To evaluate demographic differences in our groups stratified by margin status, Student’s *t*-tests were conducted for continuous variables and Chi-squared for categorical variables. A *p*-value < 0.05 was considered to be statistically significant. Multivariate logistic regression analysis was used to assess whether margin status independently predicted recurrence after adjusting for confounding variables such as age, PSA level, pathological stage, and Gleason grade group. All statistical tests and figures were conducted and produced in the R statistical package version 4.4.0 (R Foundation for Statistical Computing, Vienna, Austria).

## 3. Results

### 3.1. Patient Cohorts

A total of 159 men who underwent RARP and subsequently developed BCR were included in the study. Of these, 101 (63.5%) had NSMs and 58 (36.5%) had PSMs. Table 1 summarizes the baseline demographic and clinical characteristics stratified by surgical margin and PSMA PET/CT scan status, assuming 13 equivocal PSMA PET findings (N = 13, 8%) as positive. Corresponding analyses assuming all equivocal cases as negative are shown in Supplemental Table S1.

The median PSA at the time of positive PSMA PET scans was similar between groups (1.20 ng/mL for NSM vs. 1.37 ng/mL for PSM); however, the mean PSA levels were higher among men with PSMs (4.62 ± 10.1 ng/mL vs. 2.71 ± 8.38 ng/mL). The mean time from surgery to PSMA PET imaging was significantly shorter in patients in the PSM groups (3.21 ± 3.10 years) compared to the NSM group (7.23 ± 4.71 years; *p* < 0.001). No significant differences were observed in mean age at surgery, age at PSMA scan, or PSA levels at imaging. Among patients with PSMs, the distribution of overall Gleason grade group (GGG) was GGG-2 in 15.0%, GGG-3 in 45.0%, GGG-4 in 15.0%, and GGG-5 in 25.0% of cases (Table 1).

### 3.2. PSMA PET Findings by Margin Status

**Figure 1:**
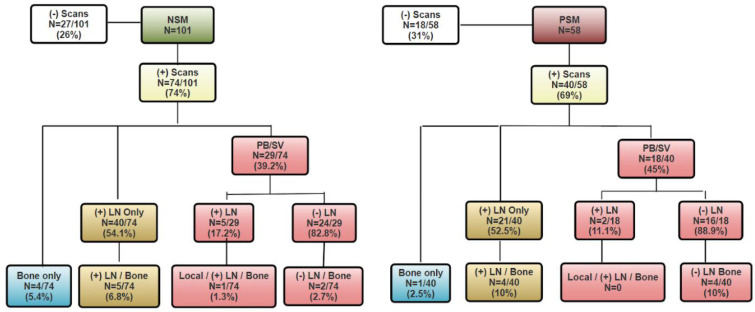
Flowchart of PSMA PET scan results by margin status.

Assuming all equivocal PSMA scan findings were positive, 73% (74/101) of men with NSMs and 69% (40/58) of those with PSMs demonstrated at least one PSMA-avid lesion (*p* = 0.56). Figure 1 depicts the distribution of PSMA PET findings by margin status. Among NSM patients with positive scans, local recurrence in the prostate bed or seminal vesicle (PB/SV) region was detected in 39.2% (29/74), lymph node (LN) involvement was detected in 60.8% (45/74), and bone lesions were detected in 16.2% (12/74). Similarly, in the PSM group, PB/SV recurrences were observed in 45.0% (18/40), LN involvement was observed in 57.5% (23/40), and bone metastases were observed in 22.5% (9/40) (Figure 1). None of these differences were statistically significant (local: *p* = 0.55; LN: *p* = 0.73; bone: *p* = 0.41). The number of lesions was also compared between the groups: single lesions occurred in 59.5% of NSM and 52.5% of PSM cases, whereas multiple lesions were observed in 40.5% and 47.5%, respectively. Pathological staging revealed a significantly higher rate of advanced disease (pT3/T4) among men with PSMs (87.5% vs. 58.1%, *p* = 0.0013), while pathological GGG distribution did not differ significantly (*p* = 0.133). Multivariate logistic regression analysis demonstrated that margin status was not a significant predictor of local recurrence (OR 1.40, 95% CI [0.65–1.54], *p* = 0.382), nor were age, p-GGG, or p-stage (Table 2).

Similar results were found (Supplemental Table S1) when assuming all 13 “equivocal scans” as “negative.” The proportion of positive scans was nearly identical between NSM and PSM cohorts (63.4% vs. 63.8%, *p* = 0.96). Similarly, rates of local recurrence (56% vs. 54%), nodal involvement (59% vs. 57%), and bone lesions (18.8% vs. 24.3%) remained statistically not significant.

### 3.3. Pathological Stage Subgroup Analysis

To further explore the relationship between pathological stage and recurrence patterns, a subgroup analysis was conducted by p-stage (Table 3). A total of 56 of 159 men (35.2%) had organ-confined pT2 disease and 103 (64.8%) had extraprostatic (pT3/T4) disease. PSMs were more frequent in the pT3/T4 group (48/103; 46.6%) compared with pT2 (10/56; 17.8%).

Among all patients, local recurrence rates did not differ significantly by p-stage (pT2 (20/56) 35.7% vs. pT3+ (27/103) 26.2%; *p* = 0.63). When stratified by both margin and stage, local recurrence occurred in 32.6% (15/46) NSM vs. 50% (5/10) PSM among pT2 cases (*p* = 0.363) and in 25.5% (14/55) NSM vs. 27.1% (13/48) PSM among pT3+ cases (*p* = 0.593). Lymph node metastases were more frequent in higher-stage disease (pT3+ 53.4% (55/103) vs. pT2 28.6% (16/56); *p* = 0.0045), while bone lesions did not differ significantly (pT2 8.9% (5/56) vs. pT3+ 15.5% (16/103); *p* = 0.24). When stratified by both margin and stage, lymph node metastases occurred in 32.6% (15/46) NSM vs. 10% (1/10) PSM among pT2 cases (*p* = 0.155) and in 54.5% (30/55) NSM vs. 45.8% (22/48) PSM among pT3+ cases (*p* = 0.380). For pT2 disease, NSM patients demonstrated a longer time interval from surgery to PSMA scan compared to PSM (8.2 ± 4.3 years vs. 3.2 ± 1.9 years; *p* < 0.001). Multivariate regression analysis showed that p-stage status was not a significant predictor of local recurrence (*p* = 0.374), nor were age, margins, or p-GGG.

## 4. Discussion

In this study, we assessed the risk and location of recurrence using PSMA-PET/CT as the detection method [20]. Given “equivocal findings” yielded by PSMA-PET, we first did testing assuming all equivocal scans as positive. In this assumption, recurrence rates were similar between men with NSM versus PSM (73% vs. 69%, *p* = 0.56). When we assumed equivocal scans as negative, the results were the same; recurrence rates did not differ between NSM and PSM (63.4% vs. 63.8%, *p* = 0.96).

Traditionally, the oncologic rationale is straightforward: when cancer extends to the surgical margin, residual disease may lead first to local recurrence and eventually to metastatic spread. Conversely, disease can also progress through lymphovascular invasion [21], leading to distant metastasis without an intervening local recurrence. Under this model, a negative margin should correspond to a low risk of local recurrence, whereas a positive margin should almost certainly signal persistent cancer and, therefore, a high likelihood of recurrence. This framework has historically justified the use of adjuvant therapies aimed at eradicating presumed residual local disease to prevent worse oncological outcomes [22,23].

However, despite decades of emphasis on the oncologic importance of achieving negative margins, our PSMA-PET-based analysis did not show a statistically significant difference in either local or metastatic recurrence between patients with negative versus positive margins. This finding persisted even when equivocal PSMA scans were treated as positive, suggesting that margin status alone may be a less reliable predictor of recurrence in the modern PSMA-PET era than previously assumed.

Many prior studies have linked PSMs with adverse oncologic outcomes [24,25,26]. However, more recent evidence complicates this traditional view. In 2023, Pellegrino et al. [15] analyzed 8141 men and found that unifocal PSMs, regardless of length, were associated with a two-fold increase in BCR at 10 years (30% for PSM vs. 15% for NSM). A logical interpretation is that 15% of NSM patients who recurred likely did so because of a biologically aggressive disease that disseminated via lymphatic or vascular pathways rather than local failure. For men with PSMs, Pellegrino et al. noted that the majority of men with PSMs did not experience recurrence despite pathological evidence of residual disease. This observation supports the interpretation that only a subset of recurrences in PSM patients represents true local failure, with the remainder driven by pre-existing systemic disease. This aligns with modern analyses showing that multifocality and Gleason group at the margin matter more than margin positivity alone [27].

Yet this interpretation leads to an unresolved question: if residual tumor is present at the inked surface, why did 85% of men with pathological evidence of residual disease at the margin not develop local recurrence? This discrepancy suggests that not all margin-positive tumor foci behave as viable, proliferative residual disease; some may be biologically quiescent or clinically inconsequential. Our results align with this evolving perspective and extend it by demonstrating that, even with modern PSMA-PET/CT—the most sensitive modality for detecting both local and distant recurrence—margin status did not correspond to higher recurrence rates in our cohort.

These findings are undeniably unexpected. Surgical doctrine has long held that achieving a negative margin is essential, because any residual tumor at the inked edge should logically translate into higher rates of local recurrence [28]. Yet in our cohort, men with positive margins did not experience higher rates of PSMA-detected local recurrence, and men with negative margins recurred at nearly the same frequency. This raises two questions: why are local recurrence rates not substantially higher in men with PSMs, and why do men with NSMs demonstrate comparable rates of local failure? Our data suggest that local recurrence may not be as tightly linked to the notion of “incomplete tumor excision” as traditionally believed. When we examined PSMA-PET-identified local recurrence in the prostate bed or seminal vesicle region—and treated all equivocal scans as positive—the incidence was 39.2% for NSMs and 45% for PSMs (*p* = 0.55). Under the opposite assumption, treating equivocal scans as negative, local recurrence rates remained nearly identical (43.8% vs. 45.9%, *p* = 0.80). Regardless of the results of equivocal scans, the conclusion remains that margin status does not meaningfully predict local recurrence in the PSMA era.

Furthermore, our study also examined PSMA-PET-detected metastatic recurrence in lymph nodes and bone and found no meaningful difference between margin groups, regardless of how equivocal scans were classified. These patterns suggest that local, nodal, and osseous recurrences may arise through similar lymphovascular mechanisms rather than from a stepwise cascade beginning with residual tumor at a positive margin, followed by local recurrence and progression to lymph node and bone metastases. The comparable rates of local recurrence in NSM and PSM patients argue against incomplete resection as the dominant cause of local failure. Instead, they point toward alternative drivers, such as early lymphovascular dissemination or microenvironmental resistance, as more plausible contributors to recurrence patterns. This interpretation is further supported by our findings, showing that pathological stage and Gleason group showed no difference in bone metastases between margin groups, as well as the observed parity in lymph node and bone metastases between the groups regardless of margin status.

From a clinical perspective, these findings have profound implications and further suggest that surgical strategies aimed at maximizing negative margins, often at the expense of functional outcomes [29], may not yield the oncologic benefit surgeons seek. Additionally, our study highlights the critical role of PSMA PET imaging in accurately characterizing disease recurrence. The newly advanced imaging technique allows for early and precise localization of recurrence even at low PSA levels [30], allowing for therapeutic planning and facilitating research to advance PC management. Patients and clinicians should follow the PSA screening recommendations of the European Association of Urology and their guidelines [19,31] to ensure early detection and systematic disease monitoring to improve long-term outcomes in PC patients.

This study is limited by its retrospective design and reliance on data from a single surgical center. No fixed SUV threshold was applied, reflecting real-world practice but potentially affecting reproducibility. The differences in time from surgery to PSMA PET/CT imaging may influence recurrence detection patterns as earlier imaging is more likely to identify lower-volume or localized disease, whereas longer intervals allow progression to higher-volume or metastatic disease. Despite this potential timing-related bias, no statistically significant differences were observed in recurrence patterns between patients with negative versus positive SMs.

## 5. Conclusions

This study demonstrates that surgical margin does not independently predict local or metastatic recurrence in men with BCR following RARP. The similarity in recurrence patterns between NSMs and PSMs groups, validated through high-sensitivity PSMA PET imaging, challenges the traditional notion on wider or more extensive oncological resections and supports a more balanced surgical approach, including consideration of nerve-sparing in appropriately selected patients. These findings further suggest that margin status alone may be insufficient to guide adjuvant radiotherapy decisions, highlighting the potential value of imaging-guided postoperative management strategies. In this context, earlier intervention and integration of PSMA PET/CT into postoperative surveillance, particularly in patients with rising PSA levels (approximately 0.7–1.0 ng/mL) or high-risk preoperative features, may facilitate earlier, more targeted interventions that could reduce BCR, the need for androgen deprivation therapy, or PCSM. Future prospective, multi-institutional studies are warranted to further validate these findings.

## Figures and Tables

**Table 1:** Demographics stratified by margin and PSMA scan status (assuming 13 “equivocals” are positive).

	Negative Margin		Positive Margin		*p*-Value
	Negative Scan	Positive Scan	Negative Scan	Positive Scan	NSM (+) Scan vs. PSM (+) Scan
	(N = 27)	(N = 74)	(N = 18)	(N = 40)	
	Mean (SD)	Mean (SD)	Mean (SD)	Mean (SD)	
**Age at Surgery**	62.5 (7.22)	61.0 (6.89)	62.0 (8.41)	63.3 (6.11)	0.0798
**Age at PSMA**	68.5 (8.15)	68.3 (6.36)	66.7 (7.96)	66.6 (6.51)	0.180
**Time from Sx to Scan**	5.94 (3.62)	7.23 (4.71)	4.69 (4.41)	3.21 (3.10)	**<0.0001**
Median [IQR]	5.10 [3.20–8.70]	6.95 [3.20–10.8]	3.73 [2.10–6.60]	2.20 [0.70–4.80]	
**PSA at PSMA**	0.78 (0.604)	2.71 (8.38)	2.28 (5.32)	4.62 (10.1)	0.283
Median [IQR]	0.70 [0.30–1.00]	1.20 [0.80–2.10]	0.94 [0.60–1.30]	1.37 [1.00–3.30]	
	**N (%)**	**N (%)**	**N (%)**	**N (%)**	
**Lesions**					0.476
Negative	27 (100%)	0 (0%)	18 (100%)	0 (0%)	
Single	0 (0%)	44 (59.5%)	0 (0%)	21 (52.5%)	
Multiple	0 (0%)	30 (40.5%)	0 (0%)	19 (47.5%)	
**Local Recurrence**					0.804
Negative	27 (100%)	45 (60.8%)	18 (100%)	22 (55.0%)	
Prostate bed	0 (0%)	25 (33.8%)	0 (0%)	16 (40.0%)	
Seminal vesical	0 (0%)	4 (5.4%)	0 (0%)	2 (5.0%)	
**Lymph Node**					0.732
Negative	27 (100%)	29 (39.2%)	18 (100%)	17 (42.5%)	
At least one node	0 (0%)	45 (60.8%)	0 (0%)	23 (57.5%)	
**Bone**					0.411
Negative	27 (100%)	62 (83.8%)	18 (100%)	31 (77.5%)	
At least one lesion	0 (0%)	12 (16.2%)	0 (0%)	9 (22.5%)	
**Pathological GGG**					0.133
1	1 (3.7%)	5 (6.8%)	3 (16.7%)	0 (0%)	
2	8 (29.6%)	22 (29.7%)	4 (22.2%)	6 (15.0%)	
3	11 (40.7%)	28 (37.8%)	7 (38.9%)	18 (45.0%)	
4	4 (14.8%)	7 (9.5%)	1 (5.6%)	6 (15.0%)	
5	3 (11.1%)	12 (16.2%)	3 (16.7%)	10 (25.0%)	
**Pathological Stage**					**0.0013**
pT2	15 (55.6%)	31 (41.9%)	5 (27.8%)	5 (12.5%)	
pT3/pT4	12 (44.4%)	43 (58.1%)	13 (72.2%)	35 (87.5%)	

**Table 2:** Multivariate regression finding predictors of a positive local recurrence.

Characteristic	OR	95% CI	*p*-Value
Age at Surgery	0.96	0.91, 1.02	0.170
Pathological Gleason Grade Group			
1	-	-	
2	0.96	0.21, 4.59	0.959
3	0.68	0.15, 3.27	0.612
4	0.48	0.08, 2.83	0.411
5	0.64	0.12, 3.53	0.598
**Pathological Stage**			
pT2	-	-	
pT3	0.70	0.32, 1.54	0.374
**Margins**			
Negative	-	-	
Positive	1.40	0.65, 1.54	0.382
OR = Odds Ratio; Cl = Confidence Interval			

**Table 3:** Demographics stratified by margin and p-stage (assuming 13 “equivocals” are positive).

	Negative Margin		Positive Margin		*p*-Value	
	pT2	pT3	pT2	pT3	NSM, pT2 vs. PSM, pT2	NSM, pT3 vs. PSM, pT3
	(N = 46)	(N = 55)	(N = 10)	(N = 48)		
	Mean (SD)	Mean (SD)	Mean (SD)	Mean (SD)		
**Age at Surgery**	60.2 (7.26)	62.5 (6.62)	59.8 (5.78)	63.5 (6.94)	0.871	0.456
**Age at PSMA**	68.4 (7.42)	68.3 (6.39)	63.0 (4.34)	67.4 (7.16)	**0.0313**	0.502
**Time from Sx to Scan**	8.20 (4.29)	5.78 (4.34)	3.16 (1.91)	3.78 (3.85)	**<0.001**	**0.0157**
Median [Min, Max]	8.80 [0.400, 18.6]	4.50 [0.300, 17.1]	2.98 [1.10, 7.50]	2.85 [0.192, 18.4]		
**PSA at PSMA**	3.05 (10.6)	1.47 (1.27)	2.06 (1.75)	4.28 (9.75)	0.771	**0.0366**
Median [Min, Max]	1.05 [0.260, 72.5]	1.09 [0.140, 6.60]	1.31 [0.260, 5.80]	1.14 [0.300, 57.6]		
	**N (%)**	**N (%)**	**N (%)**	**N (%)**		
**Lesions**					0.555	0.811
Negative	15 (32.6%)	12 (21.8%)	5 (50.0%)	13 (27.1%)		
Single	21 (45.7%)	23 (41.8%)	3 (30.0%)	18 (37.5%)		
Multiple	10 (21.7%)	20 (36.4%)	2 (20.0%)	17 (35.4%)		
**Local Recurrence**					0.363	0.593
Negative	31 (67.4%)	41 (74.5%)	5 (50.0%)	35 (72.9%)		
Prostate bed	14 (30.4%)	11 (20.0%)	4 (40.0%)	12 (25.0%)		
Seminal vesical	1 (2.2%)	3 (5.5%)	1 (10.0%)	1 (2.1%)		
**Lymph Node**					0.155	0.380
Negative	31 (67.4%)	25 (45.5%)	9 (90.0%)	26 (54.2%)		
At least one node	15 (32.6%)	30 (54.5%)	1 (10.0%)	22 (45.8%)		
**Bone**					0.897	0.768
Negative	42 (91.3%)	47 (85.5%)	9 (90.0%)	40 (83.3%)		
At least one lesion	4 (8.7%)	8 (14.5%)	1 (10.0%)	8 (16.7%)		
**Pathological GGG**					0.595	0.839
1	4 (8.7%)	2 (3.6%)	2 (20.0%)	1 (2.1%)		
2	17 (37.0%)	13 (23.6%)	2 (20.0%)	8 (16.7%)		
3	17 (37.0%)	22 (40.0%)	3 (30.0%)	22 (45.8%)		
4	4 (8.7%)	7 (12.7%)	2 (20.0%)	5 (10.4%)		
5	4 (8.7%)	11 (20.0%)	1 (10.0%)	12 (25.0%)		
**PSMA Scan**					0.303	0.536
Negative	15 (32.6%)	12 (21.8%)	5 (50.0%)	13 (27.1%)		
Positive	31 (67.4%)	43 (78.2%)	5 (50.0%)	35 (72.9%)		

## Data Availability

The dataset generated during and/or analyzed during the current study are available from the corresponding author on reasonable request.

## Supplementary Materials

The following supporting information can be downloaded at https://www.mdpi.com/article/10.3390/cancers18010043/s1; Table S1: Demographics stratified by margin and PSMA scan status (assuming 13 “equivocals” are negative).
