# Supplementary material for: Assessment of Local and Metastatic Recurrence Following Robot-Assisted Radical Prostatectomy by Margin Status Using PSMA PET/CT Scan
Source: Cancers (Basel). 2025 Dec 23;18(1):43. doi: 10.3390/cancers18010043 (PMC12785039; doi:10.3390/cancers18010043)
Supplement: Supplementary file 1 [file cancers-18-00043-s001.zip › cancers-4022321-supplementary.pdf]

Supplemental Table

Supplemental Table S1. Demographics Stratified by Margin and PSMA Scan Status (assuming 13 "equivocals" are negative)

	Negative Margin		Positive Margin		P-value
	**Negative Scan (N=37)**	**Positive Scan (N=64)**	**Negative Scan (N=21)**	**Positive Scan (N=37)**	**NSM (+) Scan vs PSM (+) Scan**
	**Mean (SD)**	**Mean (SD)**	**Mean (SD)**	**Mean (SD)**	
**Age at Surgery**	61.6 (6.85)	61.3 (7.10)	61.9 (7.83)	63.5 (6.28)	0.121
**Age at PSMA**	68.5 (7.58)	68.3 (6.45)	66.4 (7.56)	66.7 (6.64)	0.238
**Time from Sx to Scan**	6.85 (3.97)	6.89 (4.76)	4.46 (4.17)	3.22 (3.17)	**<0.0001**
Median [IQR]	7.60 [3.30-9.60]	6.35 [3.10-10.6]	3.86 [2.10-4.80]	1.80 [0.70-4.90]	
**PSA at PSMA**	0.934 (0.69)	2.90 (9.00)	2.25 (4.91)	4.83 (10.5)	0.331
Median [IQR]	0.75 [0.40-1.20]	1.19 [0.80-2.00]	1.05 [0.60-1.60]	1.34 [1.00-3.40]	
	**N (%)**	**N (%)**	**N (%)**	**N (%)**	
**Lesions**					0.0869
Negative	37 (100%)	0 (0%)	21 (100%)	0 (0%)	
Single	0 (0%)	39 (60.9%)	0 (0%)	16 (43.2%)	
Multiple	0 (0%)	25 (39.1%)	0 (0%)	21 (56.8%)	
**Local Recurrence**					0.766
Negative	37 (100%)	36 (56.3%)	21 (100%)	20 (54.1%)	
Prostate Bed	0 (0%)	24 (37.5%)	0 (0%)	14 (37.8%)	
Seminal Vesical	0 (0%)	4 (6.3%)	0 (0%)	3 (8.1%)	
**Lymph Node**					0.798
Negative	37 (100%)	26 (40.6%)	21 (100%)	16 (43.2%)	
At least 1 Node	0 (0%)	38 (59.4%)	0 (0%)	21 (56.8%)	
**Bone**					0.508
Negative	37 (100%)	52 (81.3%)	21 (100%)	28 (75.7%)	
At least 1 lesion	0 (0%)	12 (18.8%)	0 (0%)	9 (24.3%)	
**Pathological GGG**					0.191
1	3 (8.1%)	3 (4.7%)	3 (14.3%)	0 (0%)	
2	11 (29.7%)	19 (29.7%)	5 (23.8%)	5 (13.5%)	
3	15 (40.5%)	24 (37.5%)	9 (42.9%)	16 (43.2%)	
4	4 (10.8%)	7 (10.9%)	1 (4.8%)	6 (16.2%)	
5	4 (10.8%)	11 (17.2%)	3 (14.3%)	10 (27.0%)	
**Pathological Stage**					**0.003**
pT2	19 (51.4%)	27 (42.2%)	5 (23.8%)	5 (13.5%)	
pT3/pT4	18 (48.6%)	37 (57.8%)	16 (76.2%)	32 (86.5%)	
